# Obtaining the best results: aspects of data collection, model finalization and inter­pretation of results in small-mol­ecule crystal-structure determination

**DOI:** 10.1107/S2056989020005368

**Published:** 2020-05-01

**Authors:** Anthony Linden

**Affiliations:** a Department of Chemistry, University of Zurich, Winterthurerstrasse 190, CH-8057 Zurich, Switzerland

**Keywords:** structure determination, reciprocal lattice layer images, twinned data, disorder modelling, use of restraints, validation, inter­pretation of results, crystal structure

## Abstract

In small-mol­ecule single-crystal structure determination, we now have at our disposal an inspiring range of fantastic diffractometers with better, brighter sources, and faster, more sensitive detectors. Faster and more powerful computers provide integrated tools and software with impressive graphical user inter­faces. Yet these tools can lead to the temptation not to check the work thoroughly and one can too easily overlook tell-tale signs that something might be amiss in a structure determination; validation with *checkCIF* is not always revealing. This article aims to encourage practitioners, young and seasoned, by enhancing their structure-determination toolboxes with a selection of tips and tricks on recognizing and handling aspects that one should constantly be aware of. Topics include a pitfall when setting up data collections, the usefulness of reciprocal lattice layer images, processing twinned data, tips for disorder modelling and the use of restraints, ensuring hydrogen atoms are added to a model correctly, validation beyond *checkCIF*, and the derivation and inter­pretation of the final results.

## Abstract 2

This article aims to encourage practitioners, young and seasoned, by enhancing their structure-determination toolboxes with a selection of tips and tricks on recognizing and handling aspects of data collection, structure modelling and refinement, and the interpretation of results.

## Introduction   

This article arose out of my presentation entitled *Seeing is Believing: Model Finalisation and Inter­pretation of Results* given during the microsymposium *Teaching New Dogs Old Tricks* held at the 32nd European Crystallography Meeting, Vienna, August 21, 2019. One objective of this educational microsymposium was to pass on knowledge about aspects of small-mol­ecule single-crystal structure determination to younger practitioners. Two other articles arising from the microsymposium have already been published in this journal by Clegg (2019 ▸) and Spek (2020 ▸). The rationale for the microsymposium has been described eloquently in the introduction to the Clegg paper.

In small-mol­ecule single-crystal structure determination, instrumentation and user-friendly software and tools for collecting data, then solving, model-building and refining crystal structures have advanced tremendously in recent times, way beyond what one could have imagined 30+ years ago, and such developments can be expected to continue: for example, the rekindled inter­est in electron diffraction over the last couple of years, with the promise of dedicated electron diffractometers already on the horizon. We now have at our disposal an inspiring range of fantastic diffractometers with better, brighter sources, and faster, more sensitive detectors. Data collections from smaller crystals than ever before are routinely feasible. Faster and more powerful computers provide integrated tools and software with impressive graphical user inter­faces (GUIs). However, these tools should be used well, wisely and conscientiously! With a nice GUI, there is great temptation to click quickly through all the steps of, for example, a structure refinement and the task is complete within minutes, but is the result correct? Is it the best possible result? How can one be sure? Nothing is foolproof (yet!) and users should not rely 100% on automated tools. The trap with these nice GUIs is that one tends to forget to look carefully through the output results, such as the *.lst file produced by *SHELXL* (Sheldrick, 2015*b* ▸), and other information that might not be visible on the current GUI screen (or even might actually be displayed, but overlooked) to ensure that there is not an indication that something is amiss. One must always consider the chemical logic of the structural model, especially if it turns out to be an unexpected compound. New structural features, such as an unusual bond length, are quite rare these days and claims about such things often have an erroneous crystal structure behind them. Publishing a wrong structure can be at best embarrassing, at worst career destroying. The sincere advice here is: resist the temptation to click, click, click without the accompanying check, check, check as you go.

Some software advances involved simply taking a lot of the work we used to have to do manually, such as hand-editing files or poring over difference maps on line-printer paper, and making a nice GUI, which allows us to achieve the same ends with a few quick clicks of the mouse. Now, even fully automated structure solution and refinement routines are available, which, in many cases, do a handsome job of producing an almost finished structure model. Yet, there are many cases where the automated routines will, quite understandably, not quite get it right, or additional tweaking is required to obtain the best and most appropriate model for a structure. Disorder, twinning and other non-routine things in a crystal or in the data may fool the automatics, so that the user still must remain observant and finalize things manually. In my experience, no two structure determinations are entirely alike: many ‘special features’ can appear maybe once in 50 or more structures, so we need an extensive toolbox of tricks, knowledge and experience to fall back on.

Newcomers to crystal-structure determination can often learn some of the needed background theory and practice from textbooks (although attending a dedicated course is much better!), while instrument operation procedures and model refinement techniques can be gleaned from software manuals, but these do not always mention those seat-of-pants, gut-feeling little things that an experienced person does or checks for almost automatically during every structure determination. One might successfully complete many structure determinations before encountering a seldom-occurring ‘feature’ for the first time. There is then a small risk that such a feature goes unnoticed or is ignored, because the person is simply unaware of the tell-tale signs of an unusual observation and how to treat, optimize or inter­pret it.

This article therefore aims to encourage practitioners, young and seasoned, by enhancing their crystallographic toolboxes with a selection of those aspects that one should be aware of. The discussion will include some of the ways of going beyond the automatic and push-button procedures to ensure that the final structure model is truly correct and the best possible outcome from the data at hand. Aside from validation with *checkCIF* (Spek, 2020 ▸), simply looking at and rotating the structure model on-screen can give a good impression of the quality of the results; if it looks weird, it probably is! Unusual geometry, strange-looking atomic displacement ellipsoids or inexplicable residual electron-density peaks can all be indicators that the structure model, or the reflection data, could be deficient in some way and might require additional thought. After the refinement has been completed and validated, the real objective of the study, which is to answer a scientific question that lead to the need for a structure determination in the first place, can hopefully be addressed. This involves *inter­pretation* of the results: knowing how to derive, compare and correctly evaluate useful information from the structure determination, while keeping in mind the statistical significance of any numerical differences.

It will become apparent during the ensuing discussion that certain instrumentation and software are mentioned frequently, while others are not. This in no way implies that any instrument or software is more or less suitable or capable of the task than any other. It is merely that this author uses the mentioned items in his daily work and has the most experience with them. It is possible that some other tools, with which this author is less familiar, do an even better job.

## Aspects of data collection and data reduction   

### Data collection   

As an example of a not altogether uncommon situation, consider the case of a monoclinic crystal with a β angle very close to 90°, let’s say β = 90.2°. Based on just a few initial frames, the diffractometer software might decide that this unit cell is ortho­rhom­bic and automatically collect sufficient data for ortho­rhom­bic. Subsequently, the user discovers that it is impossible to find a suitable ortho­rhom­bic space group or solve the structure. On changing to monoclinic, the user finds that the structure solves and refines well, yet the data are incomplete, because a needed part of the monoclinic data was not collected in the ortho­rhom­bic strategy. At best a fresh data collection is needed, at worst the crystal has already been removed from the instrument and discarded or it subsequently decomposed. If it was the one and only good crystal, then that is unfortunate indeed. This problem can be avoided as follows. Look carefully at the initial unit-cell parameters during the data-collection strategy set-up, although their precision at this stage might not allow one to see very small deviations from 90°. If there are sufficient initial frames to allow a rough *R*_int_ value to be calculated, this might give an indication of the correct Laue class. Derive the expected number of mol­ecules in the unit cell, *Z*, from the 18 Å^3^ per atom rule (for organics and organometallics) and the unit-cell volume and see whether or not *Z* is compatible with possible crystal systems and/or space groups. If there is doubt or, in particular, not enough room in the unit cell for the minimum *Z* required by the higher symmetry, choose the lower symmetry crystal system in order to ensure enough unique data are collected, or even collect at least a full hemisphere of data to be absolutely sure. Even if the higher symmetry proves to be correct in the end, having more than enough data is always better than insufficient data, and data collections are so rapid these days that the extra data-collection time hardly has an impact on the laboratory schedule. The calculation of *Z* can be misleading, or inconclusive, of course, when there are multiple mol­ecules in the asymmetric unit (*Z′* > 1), or the compound is very different from that expected, or there are significant qu­anti­ties of solvent mol­ecules in the structure.

High redundancy in the data, such as at least fivefold, is normally recommended anyway, so as to enable good absorption corrections when using the spherical harmonics method (sometimes known as the multi-scan or empirical method) incorporated in programs such as *SADABS* (Krause *et al.*, 2015 ▸) or *CrysAlis PRO* (Rigaku Oxford Diffraction, 2019 ▸). There is now a validation alert in *checkCIF*, which is activated when the multiplicity of measurements is lower than advisable.

When one obtains data through the kind auspices of a colleague or service facility at another institution, it is sometimes difficult to go back and ask the person who collected the data for more data, or even to reprocess the existing data if what you received originally was not optimal. Nevertheless, it is disappointing if one has to try to publish a structure with the sub-optimal data. I can only suggest trying to establish a good rapport with the remote facility at the outset and discuss clearly your specific data-collection needs for each sample, so that they can deliver the required data first time as often as possible and appreciate the situation when the occasional request for additional things arises.

### Data reduction   

**Figure 1:**
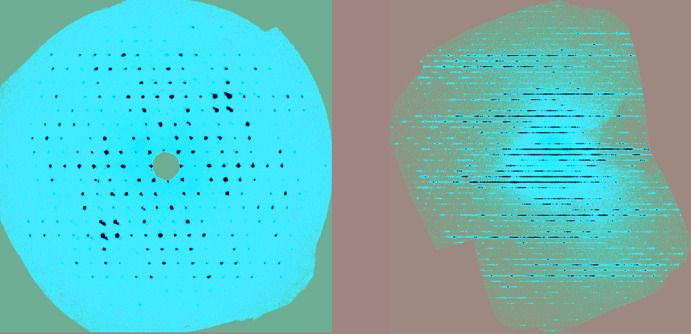
Reciprocal lattice layer reconstructions showing (left) a regular array of diffraction spots as obtained with a normal single crystal, and (right) severe streaking or rods of diffuse scattering as a result of layer stacking disorder.

It is recommended that, at the end of every data collection, one generates and inspects the reciprocal lattice layer reconstructions (also known as synthetic precession images or unwarping) of at least the *hk*0, *h*0*l*, and 0*kl* layers (Fig. 1 ▸) and more layers if more certainty is needed. These images will reveal much about the quality of the diffraction pattern, such as the presence of twinning, streaks, diffuse scattering, satellite reflections, a split crystal or just a plain poor crystal. It is important to examine all three principal plane directions, because some unexpected features might only appear in one of them.

### Twinning   

**Figure 2:**
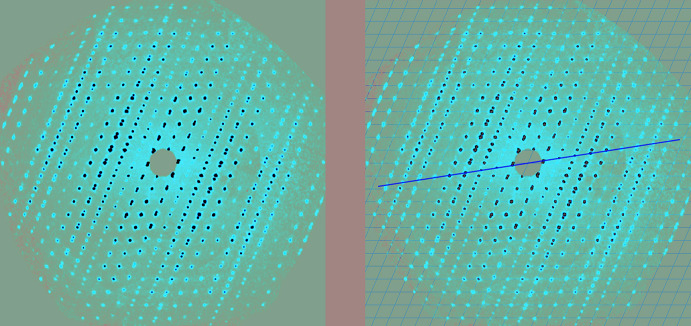
Reciprocal lattice layer reconstructions showing (left) the tell-tale sign of twinning with the overlapping, then non-overlapping, then overlapping reflections in different columns, and (right) a yellow line added as a guide to the alignment of the second twin domain.

If you discover that you have a non-merohedral or pseudo-merohedral twin, *i.e*. not all reflections from both twin domains overlap, it is best to go back to the raw diffractometer frames and look at the reciprocal lattice layer reconstructions, as mentioned above. This may be revealing, as shown in Fig. 2 ▸. If twinning is evident, the integration should be repeated with two or more orientation matrices. This will yield a reflection file that contains non-overlapping reflections from *both* twin domains plus those that are overlapping, thereby giving the most complete data set. This is the so-called HKLF5 reflection file if you are a user of *SHELXL* (Sheldrick, 2015*b* ▸). Sometimes several integration trials will be needed to find the optimal twin integration parameters, such as percentage reflection overlap when two reflections are considered individuals or not, or the use of common or separate scale factors in the finalization step.

**Figure 3:**
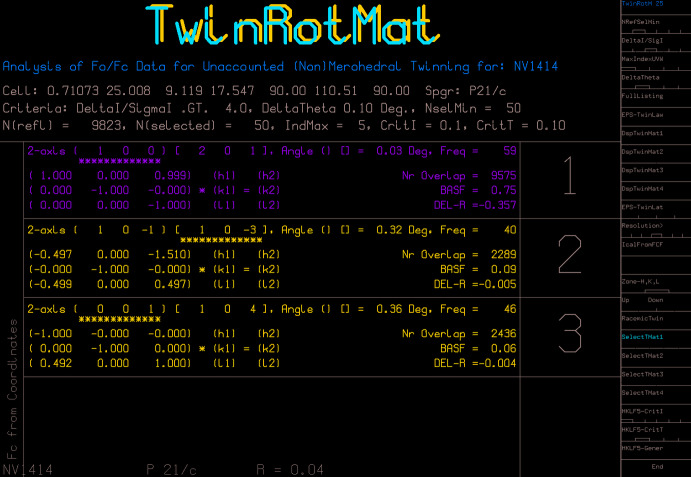
A screen from *PLATON* TwinRotMat showing the suggested best twin matrix, the twin operation, rotation about the reciprocal axis vector [100], the number of overlapping reflections, 9575 out of 9823, and the approximate major twin component of 0.75.

Sometimes, one does not realise that twinning is present until there are difficulties with the structure modelling, inexplicable residual electron-density peaks, or the refinement *R*-factor remains stubbornly high. A test of the reflection data with the *TwinRotMat* option of *PLATON* (Spek, 2020 ▸) can be revealing (Fig. 3 ▸). With this option, it is possible to produce an HKLF5 file directly from the existing standard HKLF4-type file, but in this case the HKLF5 file will contain only the overlapping reflections and the non-overlaps from one twin domain. The non-overlaps from the second twin domain are not available, because they were never included in the original HKLF4 file. While this method is a quick way to ‘get out of jail’ with unexpected twinning and achieve a reasonable final structure, ideally, one should really go back to the raw frame data and at least try to obtain the full reflection data from both twin domains to see if this is the better data set or not.

## Structure solution and refinement   

### *SHELXT* tricks   

*SHELXT* (Sheldrick, 2015*a* ▸) has proven to be an excellent tool for crystal-structure solution. It will propose the likely space group(s) and, if all goes well, in routine cases, one receives a fully or mostly complete model. It is important, though, to always check that the atom assignments are as one expects, or are consistent with the chemistry, if the compound is unknown or turns out not to be that expected.

**Figure 4:**
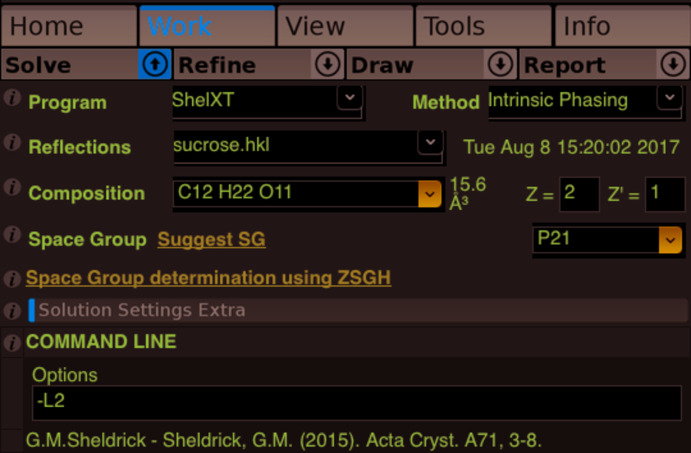
Employing the –L flag for *SHELXT* in *OLEX2*.

The space group selected by the program does, however, depend on the Laue class specified through the SYMM instructions present in the input *.ins file (the unit-cell parameters have no influence here). If these instructions are missing, only solutions in triclinic space groups will be offered. If these instructions point to the incorrect Laue class, only solutions in that Laue class will be obtained, or maybe even no solutions, such as might be the case if the program is told that the Laue class is *mmm* (ortho­rhom­bic) when in reality it is 2/*m* (monoclinic), as in the example discussed in *Section 2.1*. When starting *SHELXT*, the –L command line flag can be used to force the program to consider a Laue class other than the one specified through the SYMM instructions. For example, –L1 forces triclinic (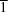), –L2 forces 2/*m*, –L3 enforces *mmm*, *etc*. The program will consider all trigonal and hexa­gonal space groups together (very handy!) with the –L15 flag. Users of *OLEX2* (Dolomanov *et al.*, 2009 ▸) can enter a –L flag in the Command Line options box of the Solve pane (Fig. 4 ▸).

*SHELXT* does not need to know the expected chemical formula, but an indication of the elements present is helpful. If the program finds heavy elements in its solution, they will be assigned to the nearest halogen by default if a heavy element has not been specified in the input instructions.

### Absolute structure, absolute configuration and the Flack parameter   

This topic has been addressed in detail already (Linden, 2017 ▸) and the reader is strongly encouraged to consult that article. Absolute structure is what is determined when one refines the absolute structure or Flack parameter (Flack & Bernardinelli, 1999 ▸, 2000 ▸). Chiral species need not be present, because the packing of the species in the crystal might still adopt a chiral or polar arrangement. If chiral species are present, one then also obtains the absolute configuration of that species. Even when no chiral species are present, a correct determination and reporting of the absolute structure is obligatory, provided the Flack parameter is precise enough.

It is important to emphasise that the precision of the absolute structure parameter [written as *x*(*u*)] must not be overlooked when deciding if the absolute structure has been determined unambiguously or not. A value of 0.01 (2) is a confident indicator, because the statistical deviation of *x* from 0.01 [0.01 ± (3 × 0.02)] is at most 0.06, so still close to zero. However, 0.0 (2) is inconclusive: at the 3σ confidence limits, the *x* parameter could statistically be 0.0 ± (3 × 0.2), so anywhere up +0.6 is possible, which is closer to 1.0 and in this case one cannot claim that the *x* parameter value of 0.0 is indicating the actual absolute structure, even when one obtains *x* = 1.0 for the inverted structure. Flack & Bernard­in­elli (1999 ▸, 2000 ▸) recommend that if a compound is known to be enanti­omerically pure, the s.u. on the Flack parameter should be less than 0.1, but if the compound *might* be present in the crystal as a racemic mixture, the s.u. should be less than 0.04.

Precise values of *x* that are neither close to 0.0 nor 1.0 are an indication of inversion twinning, where the twin components can make equal [*e.g*., *x* ≃ 0.50 (1)] or unequal contributions [*e.g*., *x* = 0.36 (1)] to the whole. In that case, *SHELXL* users must use the TWIN and BASF instructions, because without these instructions, the refinement is minimized against the ‘hand’ of the model without taking the value of the absolute structure parameter into account; it is calculated post refinement. One consequence can be a bias in the geometrical parameters. The use of TWIN/BASF takes the contributions from each ‘hand’ of the model into account in the correct proportions during refinement. This is especially important when heavy or strong anomalously scattering elements are present; a noticeable decrease in the *R*-factors can be observed compared with the standard refinement without TWIN/BASF.

Occasionally, people forget that if a mol­ecule with a stereogenic centre crystallizes in a centrosymmetric space group, or in a non-centrosymmetric space group that includes mirror, glide or roto-inversion symmetry operations, such as *Pna*2_1_ or *I* 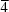, then the compound is necessarily racemic.

In *OLEX2*, a structure model can easily be inverted by typing ‘inv –f’ on the command line, which also takes care of the required change of space group if one has one of the enantiomorphic pairs of space groups, such as *P*6_1_, which would be changed to *P*6_5_.

Chemists sometimes worry if a crystal structure is obtained that indicates the crystal contains a racemic mixture of a compound, when the synthesis was supposed to have yielded an enanti­omerically pure substance. Keep in mind that the chosen crystal is not necessarily representative of the composition of the bulk material from which it was crystallized. A solution containing a 95% enanti­omeric excess might still produce one nice crystal of the racemate amongst more sludgy material containing the pure enanti­omer. The crystallographer then selects the beautiful crystal, naturally.

### Disorder modelling   

Disorder modelling can be quite laborious at times and one might even shy away from attempting to model mild cases of disorder. Do not be discouraged. *OLEX2* has an excellent range of tools available to simplify the task of modelling disorder. The authors of this program have provided extensive documentation and a video library to help with these and many other tasks in *OLEX2*; see *https://www.olexsys.org/Documentation* and *https://www.olexsys.org/Video-Library*. It can be considerably easier and more efficient if the atoms in the initial ordered model are labelled as desired and sorted *before* one starts to model the disorder.

**Figure 5:**
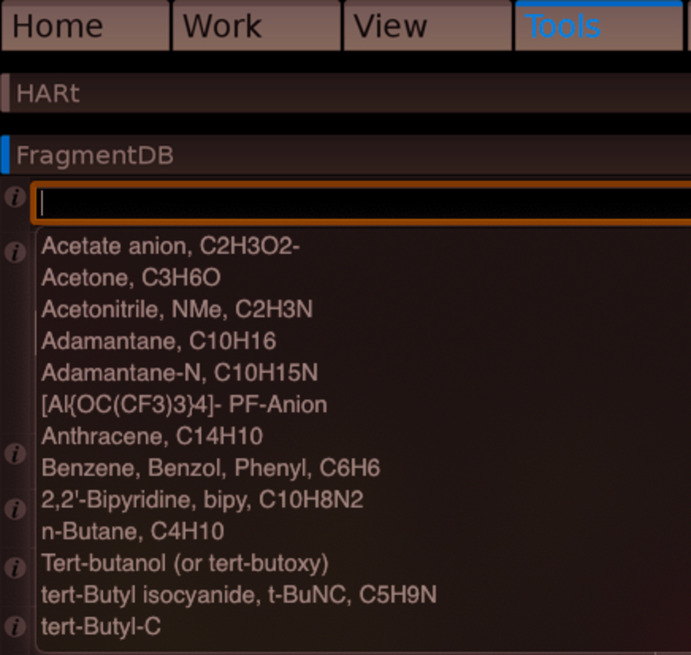
Part of the fragment database menu in *OLEX2*.

*OLEX2* also has an extensive library of pre-defined fragments and solvent mol­ecules, which can be fitted to the model under development. These can be useful for modelling complex or ill-behaved (initial) structures, whether disordered or not (Fig. 5 ▸).

There are a few things to keep in mind when modelling disorder, regardless of the software used. Do not restrain or constrain a model to be that which it is not, perhaps based on what the mol­ecule is expected to be, when in fact it is something different. One can force a model to represent any structure with enough strong restraints and constraints. If significant difference-map peaks appear on the original atom positions after you apply restraints, that might be telling you that you are pulling the model away from reality.

When developing the initial disorder model, it is best to apply geometry and displacement-ellipsoid restraints before the first refinement, as this should prevent things flying apart at the first refinement attempt. Restraints between non-bonded atoms within a group can help; for example, the F⋯F distances within a single conformation of a disordered –CF_3_ group.

Tight restraints [small standard uncertainties (s.u.s); in the *SHELXL* manual referred to as the effective standard deviation, *s*] can be effective for stabilizing the initial refinement of a disordered model. For example, one can use initial *s* values of 0.005 or 0.01, which are smaller than the *SHELXL* default values. Once things settle down, try relaxing the restraints by increasing the value of *s* or removing them altogether if things go well. In the end, the final s.u.s on restrained parameters, such as bond lengths, should be similar to those on equivalent unrestrained parameters in the model.

The *SHELXL* SAME instruction is good for larger disordered fragments, but the atoms list for the two disorder components must be in the same sequence to keep the SAME instruction simple. SIMU, DELU and/or RIGU *restraints* are preferred over EADP *constraints*, unless the linked atoms really are expected to have identical orientations of their displacement ellipsoids or these atoms share the same site. For example, the F atoms in a –CF_3_ group would not have the same displacement ellipsoid orientations, so the use of EADP there is inappropriate.

### Those tricky hydrogen atoms   

These days, good low-temperature data from good crystals of compounds not dominated by heavy elements or disorder should allow the positions of all H atoms to be detected reliably, even if most of them are subsequently placed in geometrically optimized positions. Nonetheless, finding the positions of all H atoms reliably can sometimes be a challenge, particularly with sub-optimal data quality, disordered solvent, or in the presence of heavy atoms. The addition of H atoms in geometrically idealized positions works well when the geometry and hybridization at the parent atom are well understood, such as at a methine (*sp* ^3^-hybridized CH group) or methyl­ene (*sp* ^3^-hybridized CH_2_ group) carbon atom. The HFIX and AFIX instructions in *SHELXL* are most useful in this regard. Even an instruction like HFIX 137 for adding methyl H atoms allows the group to be rotated so that it matches the underlying electron-density maxima, although this can be unreliable with poor data or disorder involving the group. Caution should be used if calculating H-atom positions when their orientation is less predictable, such as with –OH, –NH, –NH_2_ and H_2_O molecules or ligands (see below).

**Figure 6:**
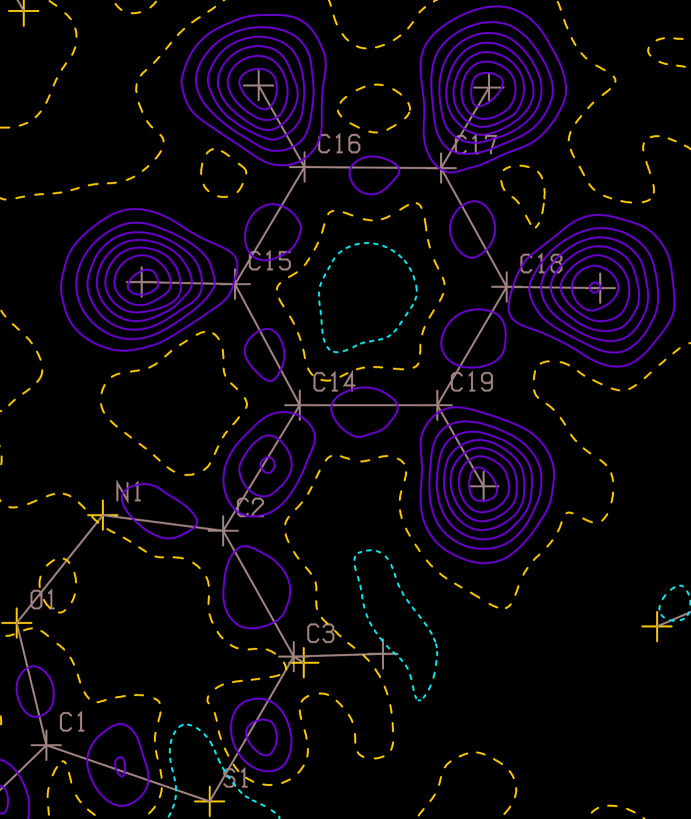
A *PLATON* *ContourDif* map with the contributions of the H atoms removed, but their positions still indicated.

It is recommended, if the data quality allows it, to try refining freely at least the H atoms bonded to heteroatoms (O, N, …); *i.e.*, position and *U*_iso_. If such H atoms refine to unrealistic positions, one can try to keep their positions fixed and refine just their isotropic displacement parameters. A good *U*_iso_ value at least indicates that the H atom is probably in the correct position. A high value suggests an incorrect position or insufficient information in the data to decide. Inspection of contoured difference maps, such as those produced with the *PLATON ContourDif* option, can also help one decide if an H atom has been placed correctly or not. The *ContourDif* function has options to display the H-atom positions, but with their contribution to the map removed, so one sees if there is a peak of electron density at each H atom site or not (Fig. 6 ▸). If there is no clear evidence to support the positions of H atoms, such as with a (disordered) solvent water mol­ecule, it is quite in order to omit such H atoms from the model, provided that the fact is documented clearly in the CIF and experimental write-up. This is better than guessing their positions just so the model and real empirical formulae match.

**Figure 7:**
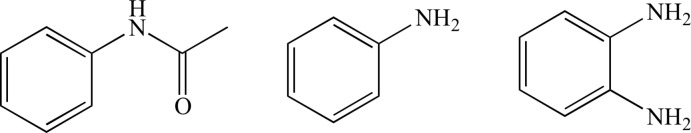
Sketches of a phenyl­amide, a phenyl­amine and a 1,2-phenyldi­amine.

Amine and amide groups need special care (Fig. 7 ▸). Amides are (almost) planar at the N atom, because of conjugation of the N-atom lone pair of electrons with the carbonyl group; in the language of organic chemists, the N atom is *sp* ^2^-hybridized. Phenyl­amines are usually planar at the N atom because of similar conjugation with the π electrons in the ring. But what about 1,2-phenyldi­amines, for example? Electron delocalization from the N atom of phenyl­amine means that the benzene ring acquires a slight negative charge (consider the classical canonical forms), but the ring is less able to accommodate additional negative charge from a second N atom of a 1,2-phenyldi­amine. There are many examples of structures in the Cambridge Structural Database (CSD; Groom *et al.*, 2016 ▸) where at least one of the amine groups in a 1,2-phenyldi­amine is somewhat pyramidalized (the N atom retains some *sp* ^3^-hybridization character), so the placement of H atoms by assuming a planar geometry with the *SHELXL* HFIX 93 instruction would be incorrect. Validation with *checkCIF* often does not detect such issues.

Programs like *OLEX2* offer a one-click button to add all H atoms to a model without having to specify the individual parent atom types. This is very convenient, but slightly risky, so it is important to inspect every atom in the model visually to ensure that the H atoms have been added or positioned correctly and none have been missed. Such automatic placement of H atoms requires the software to analyse cleverly the bond lengths and angles around every atom to which H atoms should potentially be added. The differences between certain environments can be small and where a distance or angle is inaccurate, perhaps because of data quality, variations in electron conjugation or (untreated) disorder, an incorrect choice might be made. When using *OLEX2* to add H atoms automatically, one must always check the calculated positions for –OH, –NH, –NH_2_ and H_2_O. In my experience, orienting the H atoms of such groups towards the nearest hydrogen-bond acceptor is not always the correct choice. For example, hy­droxy H atoms are sometimes incorrectly oriented and here, in particular, one should check if there is a difference electron-density peak remaining that might suggest a better orientation for the hy­droxy group. If *OLEX2* cannot decide about how many H atoms to add to a certain parent atom, those H atoms might not be included in the model, so check the model and that the empirical formula matches that expected and don’t just click on the update formula button when there is a mismatch.

**Figure 8:**
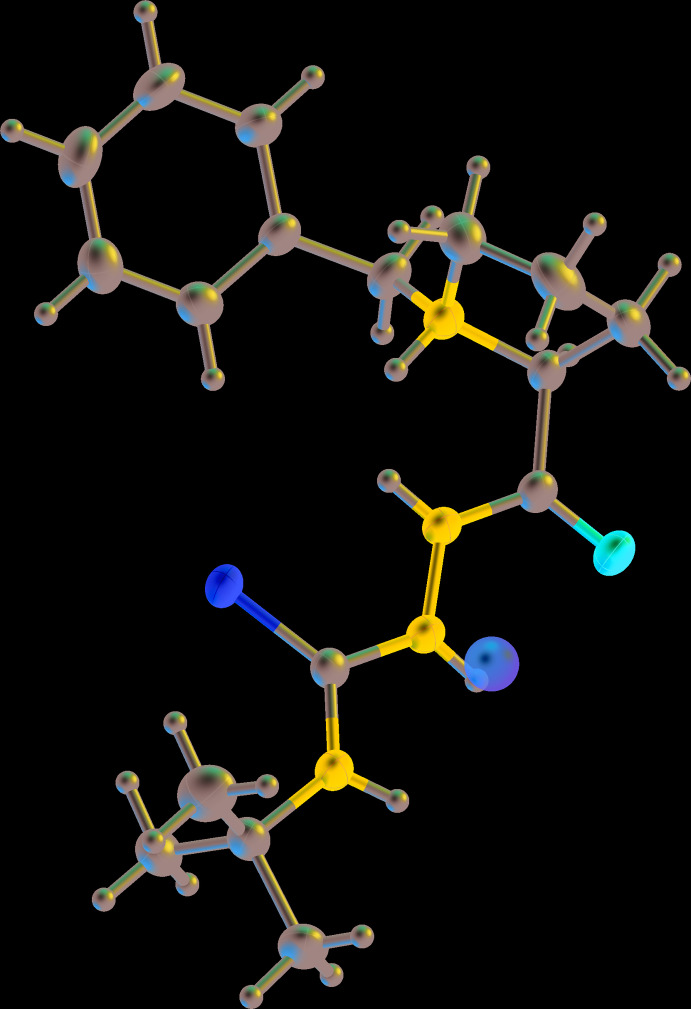
A mol­ecule after *OLEX2* has automatically added H atoms, showing an additional H atom incorrectly added to the tertiary amine N atom and a residual peak (brown sphere) near another N atom, which suggests the H atom added there assuming planar geometry is not in the best position.

The example in Fig. 8 ▸ shows what was obtained for an organic mol­ecule after H atoms were added automatically by *OLEX2* (using the version current in July 2019), but is it correct? It looks reasonable at first glance. However, an H atom has been added to the tertiary amine N atom, which would require the parent atom to be N^+^, but the mol­ecule is known to be neutral, with neither a counter-ion present nor an anionic site elsewhere on the mol­ecule. This specific mistake does not occur with the version of *OLEX2* current in March 2020, which indicates that constant improvements are being made to the algorithms for H-atom placement. In addition, the two amine groups adjacent to the S atom are not necessarily strictly *sp* ^2^-hybridized, unlike the amide N atom, so adding the H atoms assuming a planar environment might not be quite correct. Indeed, analysis of residual electron-density peaks nearby suggests that these N atoms have a somewhat pyramidal geometry indicating some *sp* ^3^-hybridization character, which is borne out if one allows all H atoms on N atoms to refine freely.

### Validation   

**Figure 9:**
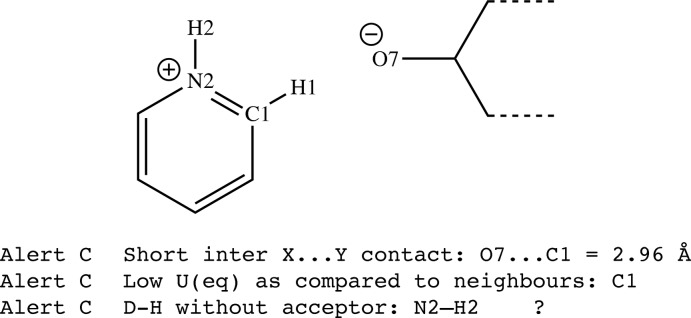
The hypothetical pyridinium cation for which atoms C1 and N2 should be exchanged.

The topic of structure validation has been discussed in detail elsewhere, most recently by Spek (2020 ▸). The IUCr *checkCIF* tool is now well established and indeed many journals require a *checkCIF* report for each structure as part of the paper submission process. While it is tempting to disregard minor validation alerts (C alerts, for example), they should always be considered. Often a small tweak to the CIF or the refinement model or strategy might resolve some of the minor alerts. Some alerts might seem unimportant when considered in isolation, because the alerted situation occurs occasionally, but, when taken together with other alerts in the list, they could indicate a real issue with the results. Consider the hypothetical case of a pyridinium cation and an anion that possesses an alkoxide group (Fig. 9 ▸). As N^+^ is isoelectronic with C, it can be difficult to be sure which atom in the ring is N^+^, although consideration of potential hydrogen-bonding inter­actions with the anion might resolve the issue. Let us say that the N atom was placed in the incorrect position, as shown in Fig. 9 ▸. Three validation alerts arise, which individually might not seem to cause much concern, but in combination more clearly point to the need to inter­change the positions of atoms N2 and C1 in the model.

**Figure 10:**
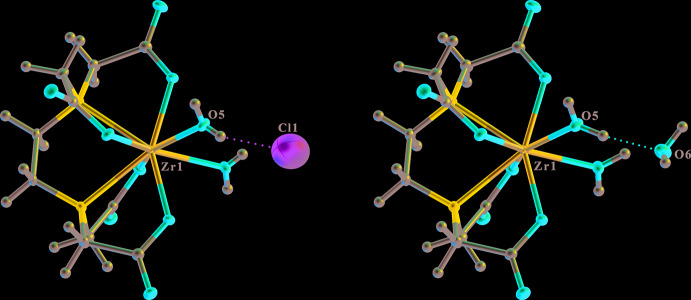
The structure of a Zr-complex with the isolated species defined (left) as a chloride ion and (right) defined correctly as a water mol­ecule.

In addition to validation, visualization and logical consideration of the structure is also important. Seeing is believing. Our eyes are great judges and a structure that visually looks strange might indeed have issues requiring attention. Ask yourself the following:

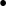
 Does the structure make sense to you, especially if the outcome is not the expected compound?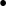 Can you rationalize the structure with the expected or plausible chemistry?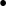 Does the structure *look right*? A clean validation report does not necessarily mean that all is in order. View a displacement ellipsoid plot from all sides. Do the atomic displacement ellipsoids look reasonable or peculiar?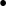 A large atomic displacement ellipsoid for an isolated atom, compared with the rest of the structure, might mean that that atom is assigned as the wrong element. In the hypothetical example of the structure of a zirconium complex, shown in Fig. 10 ▸, the chloride ion has an unusually large atomic displacement ellipsoid, but *checkCIF* does not detect this for an isolated species in the rigid-bond test, the atom-type test and the atomic displacement parameter ratio test. The *R*-factor is somewhat elevated at 0.061. The structure looks a lot better and the *R*-factor is 0.021 when the chloride ion is replaced by a water mol­ecule. The original model would require the Zr-complex to be a cation, which the chemist should realise as well.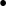 Is the mol­ecule geometrically logical and does the geometry agree with similar structures in databases? As already mentioned, geometric features not seen before for a class of compounds are rare these days and more likely to be the effect of an inadequacy in the model. Wildly varying bond lengths about a metal atom in a coordination complex have sometimes been shown to be the consequence of refining the structure in a space group without a centre of inversion, whereas in the correct structure, the metal atom lies on a centre of inversion. The near singularity (mathematical instability) of the least-squares matrix in the lower symmetry refinement is the cause of the apparent bond-length variation.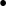 Look critically at the refinement output files, such as the *.lst file, and validation output.

Always revalidate a CIF when any changes are made to it, in case, among other things, a simple typographical mistake breaks the CIF syntax. It is ill-advised to attempt to hand-edit data in a CIF, such as reordering atomic coordinates lists, changing atom labels, *etc*. Simple typographical errors can occur easily, regardless of how careful one is. It is much better to make the necessary changes in the refinement input instructions and then run the refinement again to generate a new CIF. It is good practice with organic structures to place the C atoms first in the atom list, so that bond lengths are generated as a chemist might write them, for example, C—Br and not Br—C. For crystals containing metal atoms, they should be placed first, so bond lengths such as Fe—O rather than O—Fe are produced.

## Derivation of results   

Once a crystal structure refinement has been completed, the results can be examined in the context of the chemical question for which the determination was carried out in the first place. This is the information that might be of inter­est to a wider community than just the crystallographer. Here are some suggestions on how to derive and appropriately inter­pret the desired information.

### The use of *PLATON*   

*PLATON* is a very useful program for the derivation of results, too. Among its many functionalities is the ability to calculate just about any parameter one might wish to derive from a structure. The *Calc All* option will do much of this automatically for geometrical parameters, but there are many default settings that can be overridden if desired. For example, *Calc Coord* calculates the coordination sphere around any non-C atom out to 3.6 Å. If desired, by typing into the *PLATON* command window instead of clicking an option, one can enter the instruction with a different radius, such as *calc coord 4.5*, and all contacts out to 4.5 Å will be calculated and placed into the *.lis file.

The *PLATON* GUI has many nested option menus within several functions, like *ORTEP* or *PLUTON*, not just the one set of options initially displayed on the right. Within the menu on the right, if you see *OptionMenus* near the top, clicking between the different tick marks will bring up additional options.

Typing *help* into the initial *PLATON* window brings up a list of possible commands and their variables that one can modify.

One should always report any parameter or derived parameter with its standard uncertainty (s.u.), when available. *PLATON* calculates s.u.s for most derived parameters, but only if the CIF is used as the input file. If the *.res or *.ins file is used, s.u.s are not available to *PLATON*. Note also that if the CIF is used as input, the *PLATON NoMove* function is active so that all calculations are done with the model atomic coord­inates exactly as given in the CIF. This means that the symmetry operators for any symmetry-related atoms used in the calculations will correspond exactly with the relationships to the model coordinates. On the other hand, if the *.res file is used as input, the *NoMove* option is *inactive* by default (it can be changed by clicking on *NoMove* near the top of the menu on the right). In this case, *PLATON* sometimes optimizes the choice of asymmetric unit for the atoms before any calculations are done. The consequence for the unwary is that the reported symmetry operators for some inter­actions might not correspond with those needed when working with the original input atomic coordinates.

*PLATON* does not have access to the variance–covariance matrix generated during the least-squares refinement (Blake *et al.*, 2009*a* ▸). The consequence of this is that the s.u.s on correlated parameters can be underestimated. This may be particularly apparent when atoms on special positions or restrained H-atom positions are used in a calculation. Validation alerts about parameter s.u.s can sometimes be related to this reason. The best way to avoid this is to do the calculation, if possible, within the refinement run, because the refinement program has the variance–covariance matrix stored inter­nally during the run.

To understand how to refer to symmetry-related atoms within a *PLATON* instruction, consider the following example. If we have a slightly pyramidalized square-planar CuO_4_ moiety with the Cu atom on a twofold axis, how do we specify the atoms for a least-squares-plane calculation from which we wish to know how far the Cu atom is from the plane defined by the four coordinating O atoms? First, look at a labelled *ORTEP* diagram in *PLATON*, which automatically expands symmetric mol­ecules around symmetry sites, and note the symmetry-code letter, *a*, *b*, *c*, *etc*. appended to the label for the relevant O atoms. An alternative is to do *Calc All* and the symmetry-code letters for the expanded mol­ecule can be seen in the atomic coordinates lists near the top of the *.lis file. For the desired least-squares-plane calculation, we incorporate those symmetry code letters in the command-line instruction as follows:

lspl O1 O2 O1_a O2_a dist Cu1

### Comparing mol­ecules and structures   

Sometimes one wishes to compare the conformations of two mol­ecules in the same or different structures, or assess the degree of isostructurality between two structures. If there is more than one mol­ecule in the asymmetric unit of a structure (*Z′* > 1), these can be overlaid in *PLATON* or *Mercury* (Macrae *et al.*, 2020 ▸). Mol­ecules from different structures can be superimposed with *Mercury*, although the overlay options in *Mercury* require one to have a CSD license key.

In *PLATON*, one can use *AutoMolFit* or type ‘fit 1 2’ into the *PLATON* main window or select pairs of corresponding atoms from each mol­ecule with the *FITbyCLICK* option in the third *ORTEP* option menu. If *Z*′ > 2, mol­ecules can be overlaid pairwise with ‘fit 1 2’, ‘fit 1 3’, ‘fit 2 3’, *etc*.

**Figure 11:**
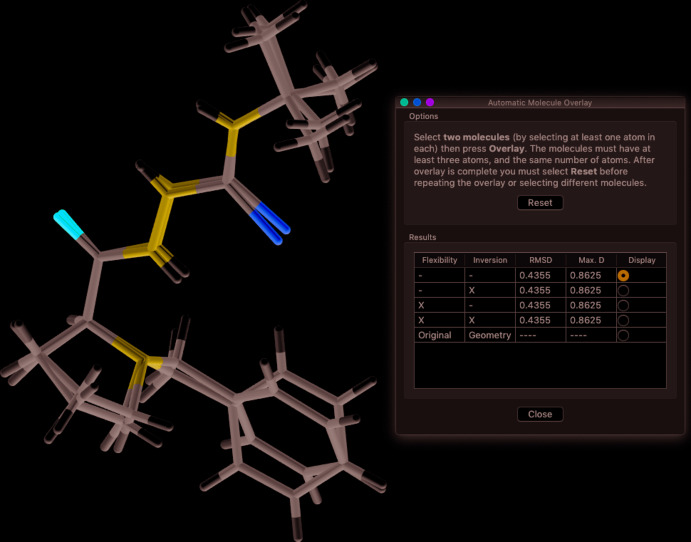
The overlay of two mol­ecules using *Mercury*. The root-mean-square deviation is also listed.

In *Mercury*, one can use the *Structure Overlay* or *Mol­ecule Overlay* items in the *Calculate* menu (Fig. 11 ▸). Alternatively, activate the *Multiple Structures* checkbox at the bottom right of the main window, *Structure Navigator* panel, which allows one to rotate the image of one mol­ecule until it aligns well with that of the other mol­ecule. When discussing the fits of the overlaid mol­ecules, one should always include the root mean square (r.m.s.) deviation of the fitted atoms so that readers know how close the fit really is.

### When are two bond lengths different?   

It can be assumed that the standard uncertainties of parameters derived from a crystal structure analysis follow a normal or Gaussian statistical distribution. This means that when comparing parameters, such as bond lengths, to find out if they are significantly different or not, one has to follow the ‘3σ rule’, which says that there is a 99.7% probability that a value belongs to a population if it lies less than 3σ from the mean of the population, where σ is the variance calculated as the value at which the height of the Gaussian curve is reduced to *e* ^−1/2^ of its maximum height found at the mean.

When comparing two crystal-structure parameters, the parameter s.u.s are equivalent to σ as defined above. The difference between the parameters is only statistically significant if it is greater than three times the s.u. of the difference. The s.u. of the difference is not the sum of the s.u.s of the two parameters, nor the mean of the two s.u.s, but the square root of the sum of the squares of the two s.u.s. In general, if two parameters are summed or their difference is calculated, the s.u. of the result is determined the same way: for *f* = *x* − *y* or *f* = *x* + *y*, σ^2^(*f*) = σ^2^(*y*) + σ^2^(*y*). For example, the C—C bond lengths of 1.523 (3) and 1.545 (4) Å have a difference of 0.022 (5) Å, so differ by 4.4σ (*i.e*., 0.022 Å = 0.005 Å × 4.4) and may be considered to be just significantly different (the s.u. of 0.005 is the square root of 0.003^2^ + 0.004^2^). The same bond lengths with larger s.u.s, such as one might find in the presence of a heavy element in the structure or slightly inferior data, could be 1.523 (7) and 1.545 (7) Å, in which case their difference is 0.022 (11) Å or 2.1σ, so based on the statistics, there is no justification for claiming that the bond lengths are significantly different in this case.

It is prudent to use caution when claiming that differences in geometrical parameters are significant when the significance level is only just greater than 3σ. It is generally accepted that s.u.s from crystal-structure refinements tend to be underestimated; reports concerning repeated determinations of the same structure suggest the underestimation is by a factor of around 1.5 to 2 (Blake *et al.*, 2009*b* ▸).

### Hydrogen bonding   

It is popular these days to analyse and describe inter­molecular inter­actions and hydrogen bonding in minute detail. It is important not to overestimate the significance of weak inter­actions when the distance is long. Ideally, calculations can be used to estimate the contribution of each inter­action, if any, to the overall lattice energy. Hirshfeld surface calculations and fingerprint plots (Mackenzie *et al.*, 2017 ▸; Spackman & Jayatilaka, 2009 ▸) are now popular means of visualizing the crystal packing and the sites of significant inter­action.

When describing inter­molecular inter­actions and the extended networks they form, it is helpful to indicate clearly which atoms or groups are involved as donor and acceptor and if the inter­action is inter­molecular or intra­molecular. Note that it is incorrect to describe hydrogen bonds between different mol­ecules or ions in the asymmetric unit as intra­molecular! One should describe how each inter­action, considered alone, links the mol­ecules or ions on an extended scale to give one aspect of the sub-structure, *e.g.* forming chains or rings, and try to indicate the direction of propagation, such as ‘chains along [100]’ or ‘planes parallel to (001)’. The square brackets convention used here refers to crystallographic vector directions, while parentheses (round brackets) refer to planes. Thus, [100] is a direction parallel to the *a* axis and should be used for 1D objects, while (001) is the *bc* plane and should be used to describe 2D objects. It is ambiguous to state, for example, that a chain propagates in the (100) plane. The use of the Etter/Bernstein graph-set motif notation can be helpful in this regard (Etter *et al.*, 1990 ▸; Bernstein *et al.*, 1995 ▸). Then, the result of the *combination* of all relevant inter­actions, such as one-dimensional chains or ribbons, two-dimensional networks or sheets, or three-dimensional networks can be stated. Some hydrogen bonds can be bifurcated [*X*—H⋯(*Y*,*Z*)] or even trifurcated [*X*—H⋯(*W*,*Y*,*Z*)].

*PLATON* and *Mercury* can be used to discover the network types and their directions, and, with the latter program, the graph-set descriptors. It is actually not difficult to do this manually for many inter­actions. For example, if a mol­ecule *A* inter­acts with *A*′ related by a 2_1_ screw axis parallel to the *c* axis, such that *A*′ is at *x*′, *y*′, *z*′ = 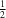 − *x*, 1 − *y*, 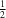 + *z*, where is the next mol­ecule, *A*′′, in this sequence? By analogy, *x*′′, *y*′′, *z*′′ = 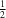 − *x*′, 1 − *y*′, 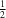 + *z*′ = 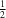 − (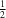 − *x*), 1 − (1 − *y*), 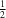 + (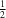 + *z*) = *x*, *y*, 1 + *z*, which is the original mol­ecule translated by one unit cell along the *z* direction, so the inter­action leads to chains running parallel to the [001] direction, even if the chains appear to have quite a zigzag form. Similar logic can be applied if *A*′ is related to *A* by a centre of inversion at the origin: *A*′ clearly lies at −*x*, −*y*, −*z* and *A*′′ is therefore at position *x*′′, *y*′′, *z*′′ = −*x*′, −*y*′, −*z*′ = −(−*x*), −(−*y*), −(−*z*) = *x*, *y*, *z*, so two iterations of the hydrogen-bonding inter­action returns us to the original mol­ecule *A*, thereby forming a ring or loop motif.

### Reporting results   

Finally, investigators usually wish to publish their results. So that a reviewer or reader of your work or a future user of your data obtained from a database can understand what you did during the structure determination, it is essential to document *fully* all non-routine procedures used during data collection and structure refinement. The final CIF does not always automatically record everything you did or your reasoning, so write it down, either in the CIF itself (preferred) or as part of the experimental section of your paper. In a CIF, the _refine_special_details or _exptl_special_details free text sections are ideal for this purpose.

Reviewers of your submitted manuscript can rightfully expect that a structure determination was routine unless told otherwise, so might query something that looks odd. The inclusion of some information about the non-routine procedure applied might satisfy the reviewer and avoid the need to ask. It does not have to be an arduous task. Simply show that you are aware of the special ‘features’ in your structure and describe briefly the techniques and tricks that you used in an attempt to address these and indicate which were successful and which were not.

This information should include details of disorder treatment, restraints and constraints used, twinning, the use of solvent masks or *SQUEEZE* (Spek, 2015 ▸) to account for disordered solvent, *etc*. For example, with a twin, one should include mention of the type of twinning, the rotation matrix relating the twin components, how the data were integrated or otherwise de-twinned, the numbers of non-overlapping reflections from each component and the number of overlapping reflections, and the refined twin fraction with its s.u. (*BASF* parameter with *SHELXL*; the s.u. is visible in the *.lst file under the details for the last refinement cycle). Depending on the software used, some of this information might already be included in the final CIF, but often these specific details are not included automatically. The same goes for describing the types of restraints applied and the tightness of the restraints, *i.e*. the effective standard deviation used.

## Conclusion   

The ongoing development of instrumentation and software for X-ray crystallography has been remarkable. Even for a relatively ‘ripe’ technique, we are still seeing significant improvement in terms of X-ray sources and detectors, and tools within software, which enable us to do things more easily and efficiently, or build better models, or even achieve results that were not thought feasible previously. Hopefully, some of the ideas and anecdotes presented in this paper will be of assistance to all practitioners in their daily efforts.

I would like to finish by expressing my heartfelt gratitude to all those out there, in companies, academic institutions or privately, who dedicate themselves to the development of instrumentation or writing the excellent software we have at our disposal today. These are eons ahead of what was available 20–30 years ago (I did my first crystal structure in 1981!). In particular, I am grateful to those who essentially voluntarily develop non-commercial software, almost as a hobby, and make it freely and openly available to the crystallographic community. Users should keep in mind that non-commercial software is rarely completely perfect or bug free, although it is jolly good nearly all of the time. When a desired feature is not available or a bug appears, please do not feel frustrated. The authors of such software always have an open ear to new ideas, requests for features and bug reports and you should not hesitate to contact them directly. This can more rapidly be productive than initiating a discussion on a forum; software developers are not necessarily monitoring all fora.

Good luck with your own structures and above all – enjoy making use of this wonderful technique!
